# Identification of a Novel Missense Homozygous Variant in *LINS1* in Two Distinct Iranian Families With Consanguineous Marriage

**DOI:** 10.1002/mgg3.70184

**Published:** 2026-02-08

**Authors:** Elham Alimoradi, Parham Nejati, Arash Salmaninejad, Nafiseh Falsafi, Fatemeh Molavi, Mohamad Javad Alibakhshi, Filippo Pinto e Vairo, Eric W. Klee, Reza Alibakhshi

**Affiliations:** ^1^ Department of Biochemistry, School of medicine Kermanshah University of Medical Sciences Kermanshah Iran; ^2^ Student Research Committee School of Medicine, Shahid Beheshti University of Medical Sciences Tehran Iran; ^3^ Medical Biology Research Center, Health Technology Institute Kermanshah University of Medical Sciences Kermanshah Iran; ^4^ Center for Individualized Medicine Mayo Clinic Rochester Minnesota USA; ^5^ Department of Clinical Genomics Mayo Clinic Rochester Minnesota USA; ^6^ Department of Medical Genetics Baran Laboratory Rasht Iran; ^7^ Department of Biology, School of Sciences Razi University Kermanshah Iran; ^8^ Dr. Alibakhshi's Medical Genetics Laboratory Kermanshah Iran; ^9^ Department of Anesthesiology Imam Reza Hospital, Kermanshah University of Medical Science Kermanshah Iran

**Keywords:** consanguineous marriage, exome sequencing, intellectual disability, *LINS1*

## Abstract

**Background:**

*LINS1*, the human homolog of the Drosophila segment polarity gene, encodes a key regulator of the Wingless/Wnt signaling pathway. While numerous genes have been implicated in intellectual disability (ID), only a limited number have been conclusively associated with autosomal recessive intellectual disability (ARID). Variants in *LINS1* have been identified as one such cause.

**Methods:**

In this study, we employed exome sequencing (ES) to investigate the genetic basis of ID in two consanguineous Iranian families. This variant was confirmed by Sanger sequencing, and segregation analysis supported its pathogenicity. Additionally, in silico analyses were conducted to explore the protein–protein interaction network of LINS1 and its functional connections to ID‐associated proteins.

**Results:**

We identified a novel homozygous missense variant in *LINS1* (c.1354G>C), leading to an alanine‐to‐proline substitution (p.Ala452Pro) in exon six.

**Conclusion:**

Our findings provide new molecular and clinical insights into the role of *LINS1* in ARID, expanding the genetic landscape of ID. This discovery has significant implications for genetic counseling and prenatal diagnosis, aiding in the identification of at‐risk couples.

## Graphical abstract

A novel homozygous missense variant in *LINS1* (c.1354G>C; p.Ala452Pro) was identified in two consanguineous families with autosomal recessive intellectual disability, supporting the gene's role in neurodevelopment and its relevance in genetic diagnosis and counseling of ARID. Reported pathogenic *LINS1* variants.

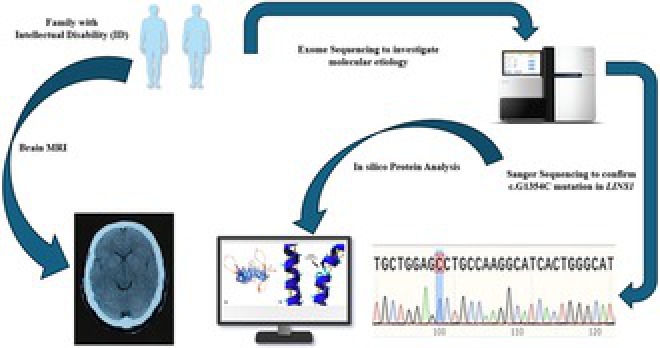

## 1 Introduction

Intellectual disability (ID), historically referred to as mental retardation (MR), presents a significant challenge for geneticists due to its diverse etiologies and complex inheritance patterns. ID is clinically defined by an intelligence quotient (IQ) below 70, accompanied by deficits in adaptive functioning and cognitive skills, manifesting before the age of 18. These criteria guide the diagnosis and management of affected individuals (Bell 1994). ID is a heterogeneous disorder with various genetic and environmental causes, including chromosomal abnormalities, monogenic mutations, and environmental factors. Monogenic ID can be further classified into syndromic and non‐syndromic forms, although distinguishing between them is often difficult. Syndromic ID is associated with additional clinical, radiological, metabolic, or biological features, whereas non‐syndromic ID primarily affects cognitive function (Szymanski and King 1999). Approximately 25% of non‐syndromic ID cases are thought to follow an autosomal recessive (AR) inheritance pattern. However, despite its prevalence, only a limited number of autosomal genes have been identified as causal for autosomal recessive intellectual disability (ARID), with most cases attributed to X‐linked inheritance. The underrepresentation of ARID genes in genetic studies is largely due to the scarcity of consanguineous families available for research (Garshasbi et al. 2008). Consequently, identifying genes responsible for non‐syndromic ARID remains a major challenge in the field. Najmabadi et al. conducted a landmark study on 136 consanguineous families, identifying 50 novel genes associated with ARID, including *LINS1*. The first pathogenic variant in *LINS1* was reported in 2011 in a family diagnosed with AR neurodevelopmental disorder (MIM: 614340) (Najmabadi et al. 2011).

**FIGURE 1:**
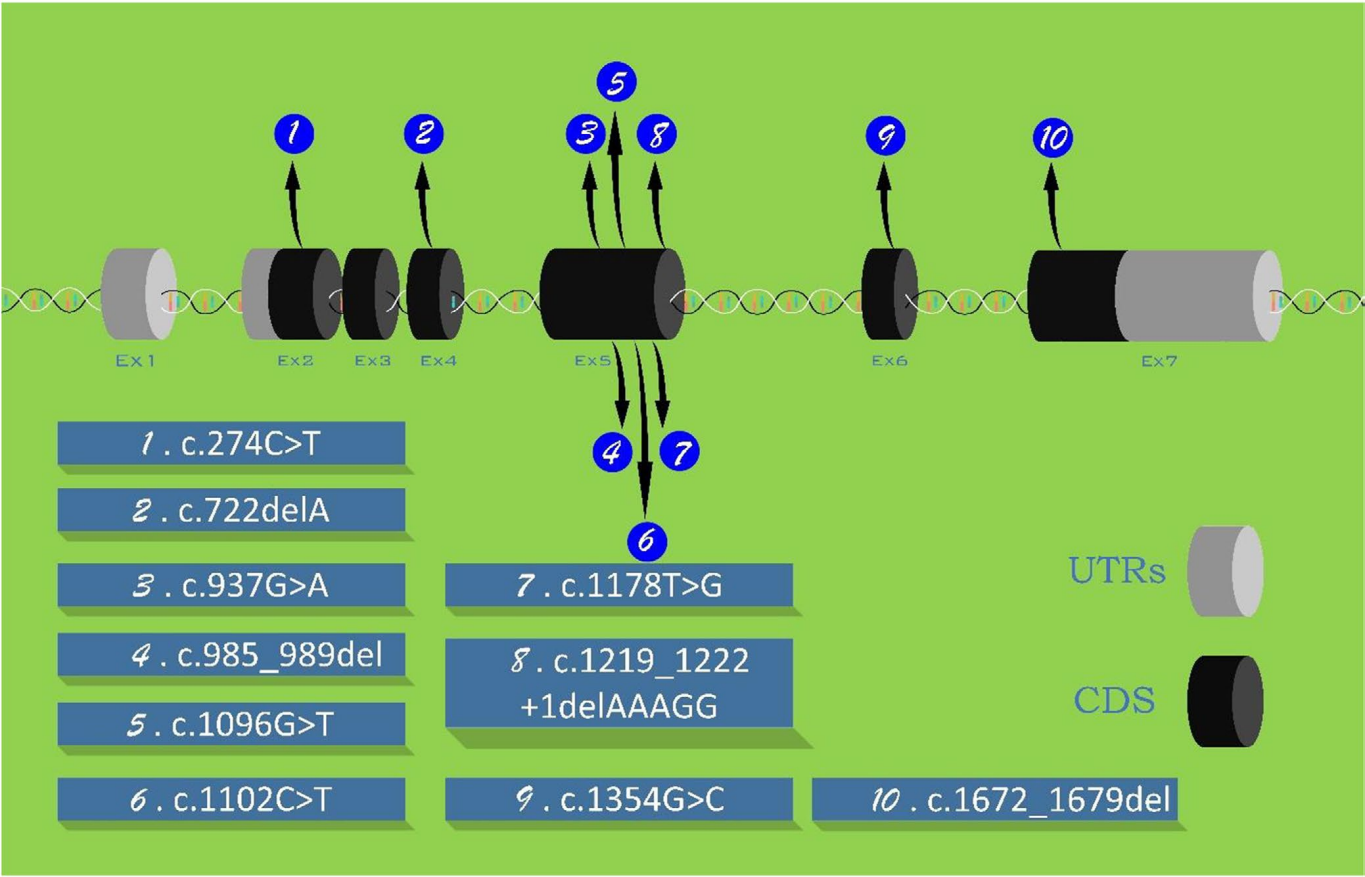
Reported pathogenic *LINS1* variants.

*LINS1* (MIM: 610350) is located on chromosome 15q26.3 and encodes a protein homologous to the *Drosophila melanogaster* segment polarity gene lin, which plays a key role in Wnt‐dependent signaling in the dorsal epidermis (Hatini et al. 2000). Despite its functional significance, only a few pathogenic variants in *LINS1* have been reported (Table 1), encompassing eleven pedigrees (27 patients) with ten causative variants (Figure 1). Current evidence suggests that *LINS1* mutations lead to disease primarily through a loss‐of‐function (LoF) mechanism, supporting its role in ARID. The increased use of genomic sequencing has substantially expanded our understanding of the genetic basis of ID. However, despite large‐scale sequencing projects, a significant number of ARID‐associated genes remain unidentified (Anazi et al. 2017; Riazuddin et al. 2017). Exome sequencing (ES) has emerged as a powerful tool for uncovering the genetic basis of heterogeneous disorders, including both syndromic and non‐syndromic forms of ID. The widespread adoption of ES has significantly increased the identification of monogenic forms of ID (Rabbani et al. 2014).

**TABLE 1:** The review of all 27 cases reported with *LINS1* pathogenic variants.

No	Variant		Number of		Zygosity	Type of mutation	Exon	Ethnicity	Phenotype	References
	cDNA (NM_001040616)	Protein	Proband	Patient						
1	c.985_989del	p.(His329*)	1	4	Hom	Frameshift	5	Iranian	Syndromic ID	Najmabadi et al. (2011)
2	c.1219_1222 +1delAAAGG	—	1	2	Hom	Splicing	5	Yemenian	ID No speech Mild flatting of mid face Hypotonia Head lag MRI normal CPK normal	Akawi et al. (2013)
3	c.937G>A	p.(Glu313Lys)	1	2	Hom	Missense	5	Gujarati Brahmins, Indian	Non‐syndromic No speech (mutism) Autistic features DD MRI normal	Sheth et al. (2017)
4	c.1096G>T c.1178T>G	p.(Glu366*) p.(Leu393*)	1	1	Compound Het	Nonsense	5	Canadian	No speech (Aphonic) ID With Wroster‐Drought syndrome MRI normal	McMillan et al. (2018)
5	c.1102C>T	p.(Gln368*)	1	2	Hom	Nonsense	5	Bangalorean, indian	Non‐syndromic Autistic features Speak delay & poor speak ID Coarse face Long tapering fingers	Muthusamy et al. (2020)
6	c.1672_1679del	p.(Gly558Pfs*22)	1	3	Hom	Frameshift	7	Afghanian	Developmental regression ID Facial dysmorphism Neurological deficits Normal MRI Flat mid face Long & thin fingers Speech impairment Hyperactive behavior	Neuhofer et al. (2020)
7	c.274C>T	p.(Gln92*)	1	2	Hom	Nonsense	2	Taiwanian	ID Speech delay Psychotic features Anxiety Schizophrenia	Chen et al. (2021)
8	c.274C>T	p.(Gln92*)	1	2	Hom	Nonsense	2	Chinese	ID Psychological symptoms Eye problems Urinary & bowel dysfunction Mitral value prolapses Q‐T prolongation	Biesecker et al. (2024)
9	c.722delA	p.(Asp241fs)	1	1	Hom	Frameshift	4	Chinese	_	Katoh (2002)
10	c.1354G>C	p.(Ala452Pro)	1	7	Hom	Missense	6	Iranian	DD ID Poor speech Autistic features Abnormal MRI Long face Flat Midface Big earlob	Current study 2025
11	c.1354G>C	p.(Ala452Pro)	1	1	Hom	Missense	6	Iranian	DD ID Autistic features Hyperactivity	Current study 2025

In this study, we aimed to further investigate the genetic etiology of non‐syndromic ARID by identifying a novel *LINS1* variant (NM_001040616:c.1354G>C; p.(Ala452Pro)) associated with ARID, detected by ES. Additionally, we conducted a comprehensive review of previously reported cases involving *LINS1* variants. *In silico* analyses were also performed to explore the protein interaction network of *LINS1* and its potential role in the pathogenesis of ID.

## 2 Material and Methods

### 2.1 DNA Extraction

Genomic DNA was extracted from peripheral blood samples of the probands and their parents using the Qiagen Flexi Gene DNA Kit. The quality and quantity of the extracted DNA were assessed using a Thermo Scientific NanoDrop One UV–Vis spectrophotometer and verified via 2% agarose gel electrophoresis. The extracted DNA was then subjected to ES.

### 2.2 Exome Sequencing and Bioinformatics Analysis

#### 2.2.1 Library Preparation and Sequencing

DNA was fragmented into 150–300 bp fragments using an ultrasonic processor, followed by target enrichment with the SureSelect Human All Exon V7 kit (Agilent Technologies, Santa Clara, CA, USA). Sequencing was performed at Macrogen (Germany) using the Illumina HiSeq 2500 platform, achieving an average coverage depth of 175×.

#### 2.2.2 Data Processing and Variant Calling

Read quality was assessed using FastQC and IlluQC. Reads were aligned to the human genome reference (GRCh38/hg38) using the Burrows‐Wheeler Aligner (BWA). Post‐alignment processing, including duplicate marking, sorting, and base recalibration, was performed using Samtools, Picard‐Tools, and BQSR (Base Quality Score Recalibration). Variants were called using the HaplotypeCaller algorithm from the Genome Analysis Toolkit version 4.0 (GATK4).

#### 2.2.3 Variant Annotation

Annotated variants were prioritized using ANNOVAR, integrating data from public and in‐house databases, including: 1000 Genomes Project (1000GP), Genome Aggregation Database (gnomAD), dbSNP, and Iranome.

#### 2.2.4 Filtering Criteria

Minor allele frequency (MAF) < 1%; presence in exonic or splice‐site regions; homozygous state (we also checked heterozygote for probable compound heterozygote); pathogenicity prediction and functional analysis; to assess variant pathogenicity, we used multiple in silico tools such as VarSome (https://varsome.com/), Franklin (https://franklin.genoox.com/clinical‐db/home), PolyPhen (http://genetics.bwh.harvard.edu/pph2/), Provean (https://www.jcvi.org/research/provean), I‐Mutant2.0 (https://folding.biofold.org/i‐mutant/i‐mutant2.0.html), MUpro (https://bio.tools/mupro) and HOPE (https://www3.cmbi.umcn.nl/hope/method/); also we used PolyPhen 2 (http://genetics.bwh.harvard.edu/pph2/), SIFT (http://sift.jcvi.org) and CADD (https://cadd.gs.washington.edu/) following the guidelines of the American College of Medical Genetics (ACMG). Additionally, STRING (https://string‐db.org/) was used for protein–protein interaction network analysis.

### 2.3 Variant Confirmation and Segregation

Sanger sequencing was used to confirm and segregate the identified variant(s) in the patient and his parents. Primers were designed and checked using Primer3 (bioinformatics.nl/cgibin/primer3plus/primer3plus.cgi) and Primer‐BLAST NCBI, respectively. Primer sequences and polymerase chain reaction (PCR) conditions were as follows: the forward primer sequence was 5′‐TGACTTACAGAGGTTCATGTC‐3′ and the reverse primer sequence was 5′‐AAAGTAAGCAACACGCCCAG‐3′. PCR was carried out in a total volume of 25 μL containing 1× PCR buffer, 1.5 mM MgCl₂, 0.2 mM of each dNTP, 0.4 μM of each primer, 1 U of Taq DNA polymerase, and 50–100 ng of template DNA. The thermal cycling conditions were as follows: initial denaturation at 95°C for 5 min; followed by 35 cycles of denaturation at 95°C for 30 s, annealing at 56°C for 30 s, and extension at 72°C for 30 s; with a final extension at 72°C for 7 min.

## 3 Results

### 3.1 Case Reports

#### 3.1.1 Patient 1

**FIGURE 2:**
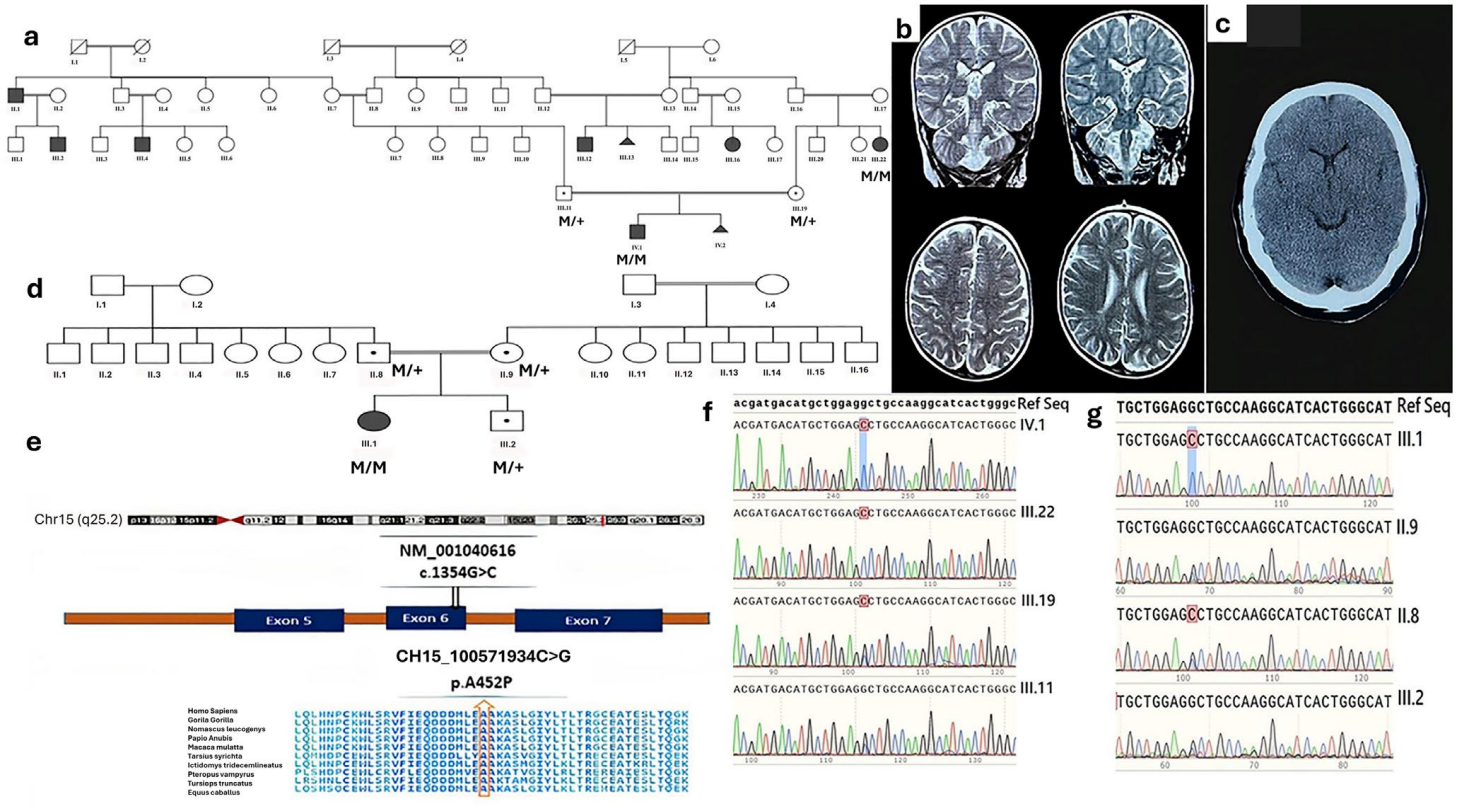
(a and d) The pedigrees for two families. This includes a segregation analysis of the identified pathogenic variant (c.G1354C; p.Ala452Pro in exon six of chromosome 15:100571934 C>G in LINS1). This mutation was found to be homozygous in affected individuals and heterozygous in their parents. The probands from Family A (IV.1) and Family B (III.1) underwent exome sequencing analysis, with genotypic data provided where available. (b and c) Brain MRI. right to left sagittal T1, axial T2, and coronal T2 scans—(c) showing the proband from Family A (IV.1), with increased signals indicating accelerated myelination near the atria in the lateral ventricles; (c) showing the proband's aunt (III.22), where all images appear normal except for altered white matter signals at the occipital horns. (e) Schematic diagram showing the location of the variant within the gene and a multiple sequence alignment of amino acid sequences across different species, highlighting high conservation of this region among species. (f and g) Sanger sequencing chromatogram. Sanger sequencing chromatogram confirms the presence of the c.G1354C; p.Ala452Pro mutation in exon six of chromosome 15:100571934 within *LINS1*. The affected patients are homozygous for this mutation, while their parents are heterozygous carriers.

An 8‐year‐old boy (Figure 2a, IV‐1), the sole child of unaffected parents from Ilam, Iran, was sent to our Genetics Clinic for evaluation. He was born to parents who are first cousins. The mother had experienced a prior pregnancy that resulted in a spontaneous abortion; however, there were no clinical or genetic details regarding the fetus (Figure 2a, IV‐2).

The proband exhibited global developmental delay (DD), significant speech impairment, poor growth, ID, and walking difficulties. Additionally, he demonstrated autistic features, including hyperactivity, impulsivity, and aggressive behavior. Exome sequencing (ES) identified a novel homozygous *LINS1* variant (NM_001040616:c.1354G>C; p.Ala452Pro). Given that his maternal aunt (Figure 2a, III‐22) exhibited similar autistic traits, sanger sequencing was performed, confirming the presence of the same *LINS1* variant.

Family history revealed multiple relatives with ID of unknown etiology, including the father's uncle and two first cousins (Figure 2a, II‐1, III‐2, and III‐4) and the mother's sister and three first cousins (Figure 2a, III‐12, III‐13, and III‐16).

Brain MRI of the proband revealed white matter abnormalities, including low signal intensity on T2‐weighted imaging (T2WI) and mild symmetric high signal intensity in the terminal myelination zones adjacent to the atria of the lateral ventricles. Other brain structures, including the lateral, third, and fourth ventricles, brainstem, cerebellar hemispheres, pituitary gland, orbits, and optic chiasm, were unremarkable (Figure 2b). In contrast, MRI of his maternal aunt (Figure 2a, III‐22) was normal except for a signal change in the white matter surrounding the occipital horn (Figure 2c). Additionally, two pregnancies in the extended family (Figure 2a, III‐13 and IV‐2) were terminated following prenatal identification of the same *LINS1* variant. No further clinical information was available for the rest of the family. Anthropometric measurements of the affected male individual from Family A revealed a head circumference of 31.7 cm, a length of 45 cm, and a birth weight of 2.6 kg at birth. At the time of the current evaluation, his head circumference measured 48 cm, with a height of 113 cm and a weight of 19 kg.

#### 3.1.2 Patient 2

The second patient, an 8‐year‐old female (Figure 2d, III‐1), was born to unaffected first‐cousin parents from Kermanshah, Iran. Upon referral to our clinic, she presented with symptoms similar to those of Patient 1, including ID, impaired gait, hyperactivity, and impulsive behavior. Notably, she had no dysmorphic features and no history of seizures.

Family history revealed that her brother was asymptomatic, and genetic testing confirmed that he was heterozygous for the *LINS1* c.1354G>C variant (Figure 2d, III‐2). Brain MRI was not available for this patient. Anthropometric measurements of the affected female individual from Family B showed a head circumference of 31 cm, a length of 42 cm, and a birth weight of 2.5 kg at birth. At the time of the current evaluation, her head circumference was 46.5 cm, height was 103 cm, and weight was 15 kg.

### 3.2 In Silico Analysis

The impacted alanine amino acid is highly conserved across species (Figure 2e). Sanger sequencing confirmed that patients were homozygous for this variant, while the parents were identified as heterozygous carriers (Figure 2f,g), establishing them as obligate carriers. Consequently, the results demonstrated the cosegregation of the c.1354G>C variant in the *LINS1* gene with the disease in the family.

**FIGURE 3:**
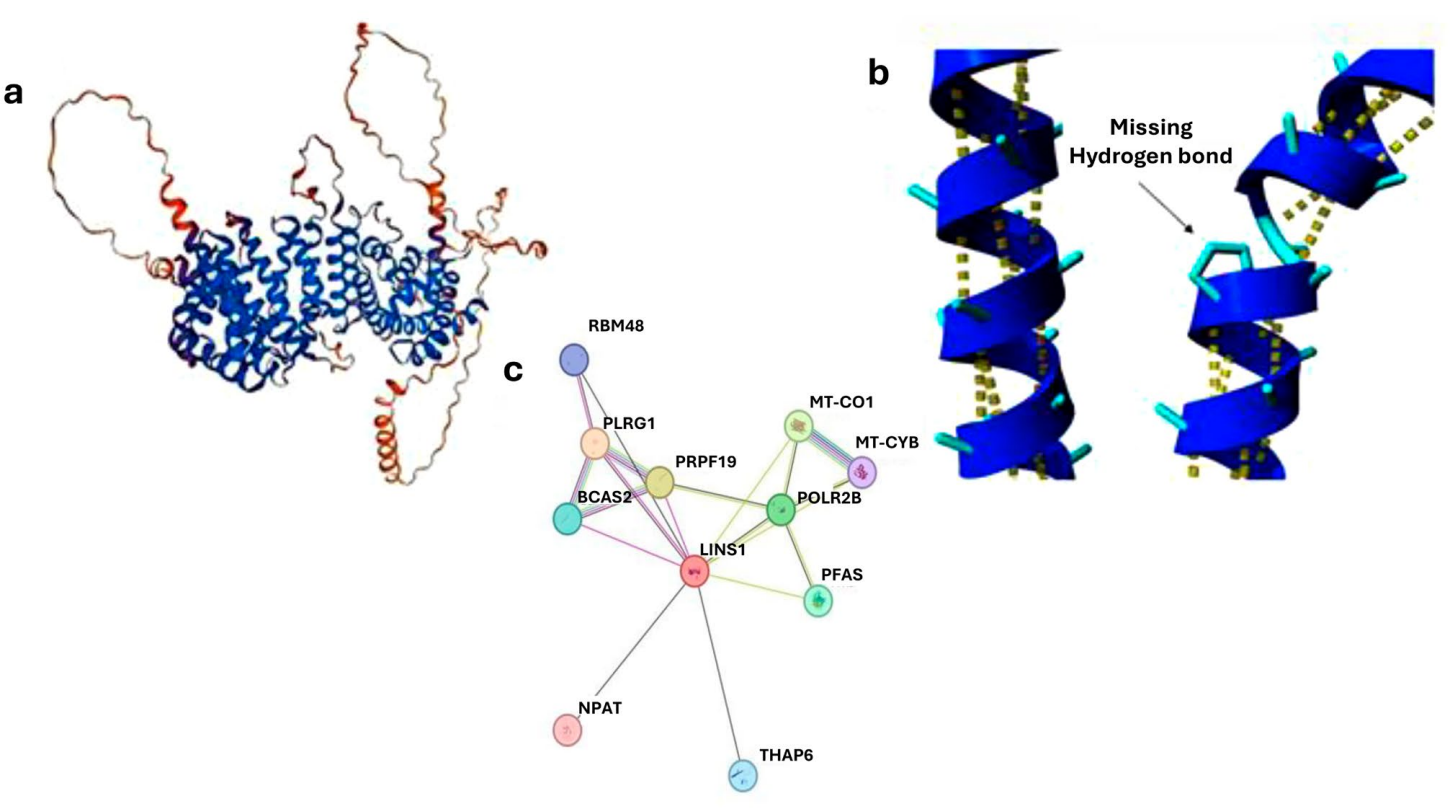
Protein structure of the variant and String network. (a) Hope prediction shows disruption of the protein folding after p.(Ala452Pro) conversion. (b) LINS1 protein structure. (c) The prediction of LINS1 association with other proteins by String online database.

The wild‐type residue is predicted to be located in an α‐helix, and the subsequent alteration of this amino acid to Proline is predicted to disrupt hydrogen bonding (Figure 3a). In fact, according to HOPE prediction, Proline disrupted an alpha‐helix when not located at one of the first three positions of that helix, and this can have severe effects on protein folding. The mutant residue was bigger in comparison to wild‐type. Therefore, assessment of the variant protein structure demonstrated changes in size, charge, and hydrophobicity. Interestingly, neither this mutant residue nor other residues at this position or homologous positions have been observed so far (Figure 3b).

This is classified as a likely pathogenic variant according to the ACMG guidelines (Richards et al. 2015). The evidence for pathogenicity includes:

PM2 supporting (+1 pt): Absent from controls (or at extremely low frequency if recessive) in population databases.

PP1 and PP4 (+5 pt): Cosegregation with disease in multiple affected family members in both families and a phenotype with locus heterogeneity.

PM3 supporting (+1 pt): homozygous variant of uncertain significance (VUS), due to consanguinity, 0.25 points towards per family with a total of 0.5 points (2 families).

Furthermore, an analysis of the interaction network of *LINS1* using the STRING database revealed strong predicted associations with several genes, including *POLR2B*, *PFAS*, *PRPF19*, *NPAT, THAP6, MT‐CO1, MT‐CYB, BCAS2, PLRG1*, and *RBM48* (Figure 3c). Several of these genes are involved in critical cellular processes. *BCAS2* and *PLRG1* function as pre‐mRNA splicing factors, while *RBM48* is associated with the minor spliceosome. *MT‐CYB* and *MT‐CO1* encode components of the mitochondrial electron transport chain, specifically cytochrome b and cytochrome c oxidase, respectively. *PRPF19* plays a role in pre‐mRNA processing and is involved in the ubiquitination of mRNA. *POLR2B* encodes a subunit of RNA polymerase II, which is essential for transcribing DNA into RNA. *PFAS* is a key enzyme in the purine biosynthetic pathway, which is necessary for DNA replication, transcription, and energy metabolism. *NPAT* encodes a nuclear protein with coactivator/corepressor activity and is involved in G1/S phase progression.

## 4 Discussion

Due to the heterogeneity of ID and the limited knowledge of the genetic basis of non‐syndromic, ARID, we performed ES on probands of two consanguineous families to identify potential existing or novel causes for their phenotype and we identified a novel variant in *LINS1*. Previous studies have shown that all patients with *LINS1* variants exhibit similar features, including ID with varying dysmorphisms. A total of ten unique variants have been identified, including ours, suggesting the allelic heterogeneity of *LINS1*‐associated ARID. (Akawi et al. 2013; Chen et al. 2021; Najmabadi et al. 2011; Neuhofer et al. 2020; Zhang et al. 2020). According to former studies, all patients with *LINS1* variants have the same similar features including NS‐ID with variable phenotypes (Table 1).

NS‐ID in young children is a neurodevelopmental disorder defined as ID with subtle neurological and psychiatric features. Although it has been challenging to differentiate between syndromic and NS‐ID, genomic sequencing is an appropriate testing strategy to identify genetic causes (Hamdan et al. 2009). Recent studies confirmed the effectiveness of ES on clinical studies of ID with unknown etiology (Kaufman et al. 2010; Rauch et al. 2012). Most studies suggest ES as a first‐line diagnostic approach for neurodevelopmental disorders, owing to the high diagnostic yield and the possibility of identifying coexistence of several disease‐causing variants (Reuter et al. 2017).

The LINS1 protein is expressed in various tissues, including the brain, prostate, skeletal muscles, and thymus (Katoh 2002). While the precise function of *LINS1* in humans remains unclear, its homolog in *Drosophila* is involved in developmental Wnt signaling. Activation of Wnt signaling facilitates the entry of the LIN protein into the nucleus, which is essential for tissue‐specific events (Hatini et al. 2000). Wnt signaling, a conserved pathway, regulates cell fate, migration, organogenesis, and neuronal development. It is divided into canonical and non‐canonical pathways, both of which play significant roles in neuron maturation and have been implicated in the pathophysiology of ID and autistic features (Kwan et al. 2016; Munji et al. 2011). It is predicted that LINS1, as a transcriptional regulator in the canonical Wnt signaling pathway, impacts downstream targets such as JAG2, EN1/2, and SMAD3, which are involved in neurogenesis and the development of the midbrain and cerebellum (Akawi et al. 2013; Hanks et al. 1995; Iwaki et al. 2001; Sander et al. 2003).

LINS1 is an evolutionarily conserved protein, and variants in conserved regions are often pathogenic. The variant p.(Ala452Pro), identified in this study, is predicted to have a damaging effect. Interestingly, the proband IV.1 of family A has a significant family history of ID, with six relatives exhibiting similar clinical phenotypes, suggesting an ARID in the family. Although ARID variants in unrelated families are rare (Kaufman et al. 2010), we observed this variant in two unrelated families who lived in different cities.

The clinical features in our probands were notably similar to those reported in previous studies, though there were some differences. For example, Chen et al. reported speech DD and moderate ID in sisters with the p.(Gln92Ter) variant located in exon 2 (Chen et al. 2021). In some cases, *LINS1* variants are associated with behavioral issues such as anxiety, hyperactivity, aggression, and autistic traits, which we also observed in our families (Akawi et al. 2013; Sheth et al. 2017). Additional clinical features in our probands, including poor growth and walking impairment, have been documented in an Indian patient with ID and the homozygous (p.Gln368Ter) at exon 5 (Muthusamy et al. 2020). Brain MRI in our probands displayed overlapping features with previous reports, including white matter changes in signal intensity, though no brain malformation or leukoencephalopathy was observed.

In accordance with the ACMG guidelines for disorders with AR inheritance and a homozygous genotype, each meiosis for an affected individual is assigned +2.0 points for the allele (Biesecker et al. 2024). For Family A, which includes seven affected individuals and three segregated individuals (excluding the parents), the total score is calculated as +6.0 points based on the affected and segregated individuals. In Family B, which has one affected and segregated individual (contributing 2.0 points) and one unaffected individual (contributing 0.4 points, excluding the parents), the total score amounts to 2.4 points. For all cases, the evidence for each locus is capped at a maximum of +5.0 points, as outlined for all variant evidence categories (e.g., PP1 and PP4) beyond this threshold (Biesecker et al. 2024). For phenotypes like ID associated with AR inheritance, where over 400 genes are implicated, a lower limit of +1.0 points for PP4 is recommended, corresponding to a diagnostic yield of approximately 20% (Ilyas et al. 2020). Additionally, when applying PM2_supporting, which adds +1.0 point based on the variant's absence or extremely low frequency in control populations (such as gnomAD and other frequency databases), the current variant reaches the required +6.0 points, classifying it as likely pathogenic. Furthermore, a supplementary +0.25 points per family can be added for PM3 (homozygous variants of uncertain significance due to consanguinity), contributing an additional +0.5 points for the two families (Oza et al. 2018).

According to network predictions, the LINS1 protein interacts with proteins associated with autosomal recessive rare diseases, including NPAT, THAP6, and MT‐CO1, all linked to neurological disorders (Bečanović et al. 2021; Herrero‐Martín et al. 2008; Mazars et al. 2010; Sang et al. 2022). Other interacting proteins such as PFAS, BCAS2, and POLR2B have been implicated in cancers (Li et al. 2023; Salmerón‐Hernández et al. 2019; Savage and Alter 2009), while PRPF19 and PLRG1 are associated with immunological diseases (Kleinridders et al. 2009; Yang et al. 2022), and RBM48 is linked to nephronophthisis. Additionally, MT‐CYB is involved in neuropathies and cardiomyopathies (Braun et al. 2016; Ryzhkova et al. 2018). To our knowledge, only PLRG1 has been experimentally confirmed to interact with LINS1, while the other proteins are predicted to interact based on network analysis. Sheth et al. suggested that LINS1 regulates ELAV1, an important protein involved in RNA binding during neuron differentiation, and that PLRG1 interacts with LINS1 for protein splicing (Sheth et al. 2017). Collectively, these findings highlight the critical role of *LINS1* and its interacting network in fundamental molecular functions. Notably, some of these genes, such as *POLR2B*, *NPAT*, and *MT‐CO1*, have been implicated in neurodevelopmental syndromes and DD, suggesting a potential link between *LINS1* dysfunction and ID (Haijes et al. 2019; Herrero‐Martín et al. 2008; Sang et al. 2022).

Further studies are needed to determine if the p.(Ala452Pro) variant is recurrent in the Iranian population at a higher‐than‐expected rate. Our patients, who are homozygous for this variant, inherited it from consanguineous parents with close birthplaces. Although the rarity of a variant is typically a prerequisite for its pathogenicity, it may represent a founder variant. Confirmation of this requires sequencing data from several cases carrying the same pathogenic variant, which is currently unavailable in the literature. Therefore, additional patient data and further in‐depth studies are necessary to verify whether p.(Ala452Pro) could be a founder variant and to explore its potential relationship with the disease.

In this study, we identified a novel homozygous variant in *LINS1* detected in two unrelated families supporting the link between *LINS1* and ARID. Our investigation also highlighted the regulatory network associated with *LINS1*, suggesting its significant role in major molecular functions, including neuronal development and growth. The diverse phenotypes observed in affected individuals further indicate broad clinical heterogeneity in *LINS1*‐related neurodevelopmental disorders. Therefore, additional molecular studies are required to elucidate the precise pathophysiological mechanisms underlying these complex clinical presentations. Our findings may contribute to future therapeutic developments, preimplantation and prenatal diagnostic programs, and genetic counseling.

## Author Contributions

E.A., P.N. and A.S. designed and performed the bioinformatics analyses, prepared the tables and figures. N.F., F.M., M.J.A. and A.S. wrote the paper. F.P.V. and E.W.K. reviewed the manuscript. R.A. supervised the study. All authors read and approved the submitted version of the article.

## Funding

The authors have nothing to report.

## Ethics Statement

This study was approved by the Research Ethics Committee of Kermanshah University of Medical Sciences, Kermanshah, Iran (Ethics code: IR.KUMS.MED.REC.1403.319).

## Consent

The patient's family has signed informed consent regarding publishing their data and their pictures.

## Conflicts of Interest

The authors declare no conflicts of interest.

## Web Resources

Varsome (https://varsome.com/), PolyPhen (http://genetics.bwh.harvard.edu/pph2/).

Franklin (https://franklin.genoox.com/clinical‐db/home).

Provean (https://www.jcvi.org/research/provean).

I‐Mutant2.0 https://folding.biofold.org/i‐mutant/i‐mutant2.0.html.

MUpro https://bio.tools/mupro.

HOPE (https://www3.cmbi.umcn.nl/hope/method/).

STRING (https://string‐db.org/).

PolyPhen 2 (http://genetics.bwh.harvard.edu/pph2/).

SIFT (http://sift.jcvi.org).

CADD (https://cadd.gs.washington.edu/).

## Data Availability

All supplementary data supporting the findings of this study are available upon reasonable request to the corresponding author.
